# Case report: a genomics-guided reclassification of a blood culture isolate misassigned by MALDI-TOF as *Yersinia pestis*

**DOI:** 10.1099/acmi.0.000422

**Published:** 2022-10-03

**Authors:** Eby M. Sim, Bryant Koh, Jimmy Ng, Trang Nguyen, Qinning Wang, Andrew N. Ginn, Mitchell Brown, David Pham, Vitali Sintchenko

**Affiliations:** ^1^​ Centre for Infectious Diseases and Microbiology- Laboratory Services, Institute of Clinical Pathology and Medical Research, NSW Health Pathology, Sydney, New South Wales, Australia; ^2^​ Sydney Institute for Infectious Diseases, The University of Sydney, Sydney, New South Wales, Australia; ^3^​ Centre for Infectious Diseases and Microbiology- Public Health, Westmead Hospital, Sydney, New South Wales, Australia

**Keywords:** genome sequencing, laboratory biosafety, laboratory identification, *Yersinia*

## Abstract

In this report, we describe a case where Gram-negative rods were isolated from a blood culture which subsequently presented a discordant *Yersinia* species result by MALDI-TOF. Rapid sequencing provided high-resolution identification of the isolate as *Yersinia pseudotuberculosis *, which was subsequently confirmed by biochemical tests.

## Case report

An 83-year-old patient originally from Turkey presented with progressive light-headedness, and dyspnoea with minimal exertion over 4 days, on a background of diabetes mellitus, congestive cardiac failure, chronic kidney disease, and hypertension. Blood tests on presentation revealed a microcytic anaemia (haemoglobin 49 g l^−1^, MCV 66 fL), which was treated with blood transfusion. The patient spiked a single fever of 38.1 °C on day six of admission for which blood was collected into BACTEC Plus Aerobic medium (BD Diagnostics) and BACTEC Lytic Anaerobic medium (BD Diagnostics). Concurrently, a non-contrast CT of chest, abdomen and pelvis was performed to investigate deranged liver function tests (with a predominantly cholestatic pattern) and hypoechoic liver lesions seen on abdominal ultrasound. The CT scan demonstrated caecal wall thickening, and endoscopic biopsies were taken. Adenocarcinoma of the proximal ascending colon without lymphovascular or perineural invasion was seen on histopathological examination. The patient proceeded to have an urgent open right hemicolectomy given ongoing transfusion-dependent blood loss. Histopathologic examination of operative tissue confirmed adenocarcinoma. Chronic inflammatory infiltrate at the advancing edge was also seen.

Gram-positive cocci and Gram-negative rods were detected in the anaerobic (17 h) and aerobic (66 h) cultures respectively and were each isolated. Reliable identification on the MALDI-TOF (MALDI Biotyper smart with a Security Library Extension; Bruker) could not be obtained due to identification discordance, with acceptable scores for *Streptococcus mitis* (score: 2.32) and *S. oralis* (score: 2.29) for the Gram-positive cocci recovered anaerobically, and *Yersinia pestis* (score: 2.25) and *Y. pseudotuberculosis* (score: 2.13) for the Gram-negative rods recovered aerobically. Viridians group streptococci were deemed not clinically significant, as trans-thoracic echocardiogram did not demonstrate a valvular vegetation and two subsequent blood cultures had no growth. However, discordant *Yersinia* results triggered a genomic analysis for identification and the patient was treated with ciprofloxacin with subsequent testing by BD Phoenix AST indicating *in vitro* susceptibility (MIC ≤0.125). Repeat blood cultures were negative.

**Fig. 1.**
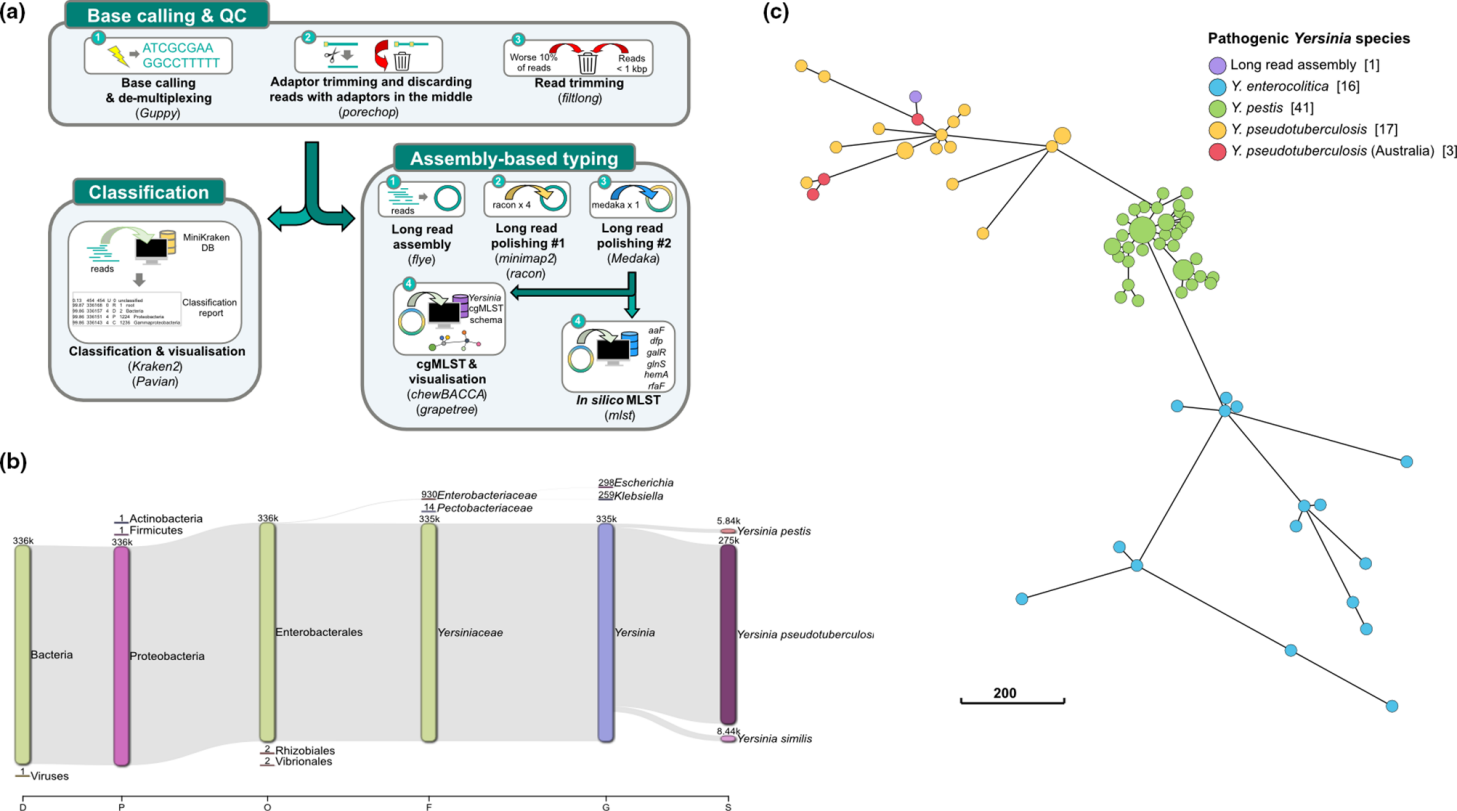
Genomic evidence for *Y. pseudotuberculosis* at the sequencing and genomic assembly level. (**a**) Graphical representation of the two-pronged approach for genomic identification of *Y. pseudotuberculosis *. Software utilised in this workflow are listed in parenthesis. (**b**) Kraken2 classification report as a Sankey plot showing the top three members in each taxonomical level. The taxonomical level on the x-axis are Domain, Phylum, Family, Genus and Species. Figure was generated using Pavian version 1.0 [17]. (**c**) Minimum spanning tree generated from 321 out of 500 loci from the *Yersinia* cgMLST scheme [7] that were present in all complete genomes in our dataset (Supplementary File 2). Each genome is represented as a node and connecting lines represent number of allelic differences, scaled according to the scale bar. Image was generated using GrapeTree [18].

**Fig. 2.**
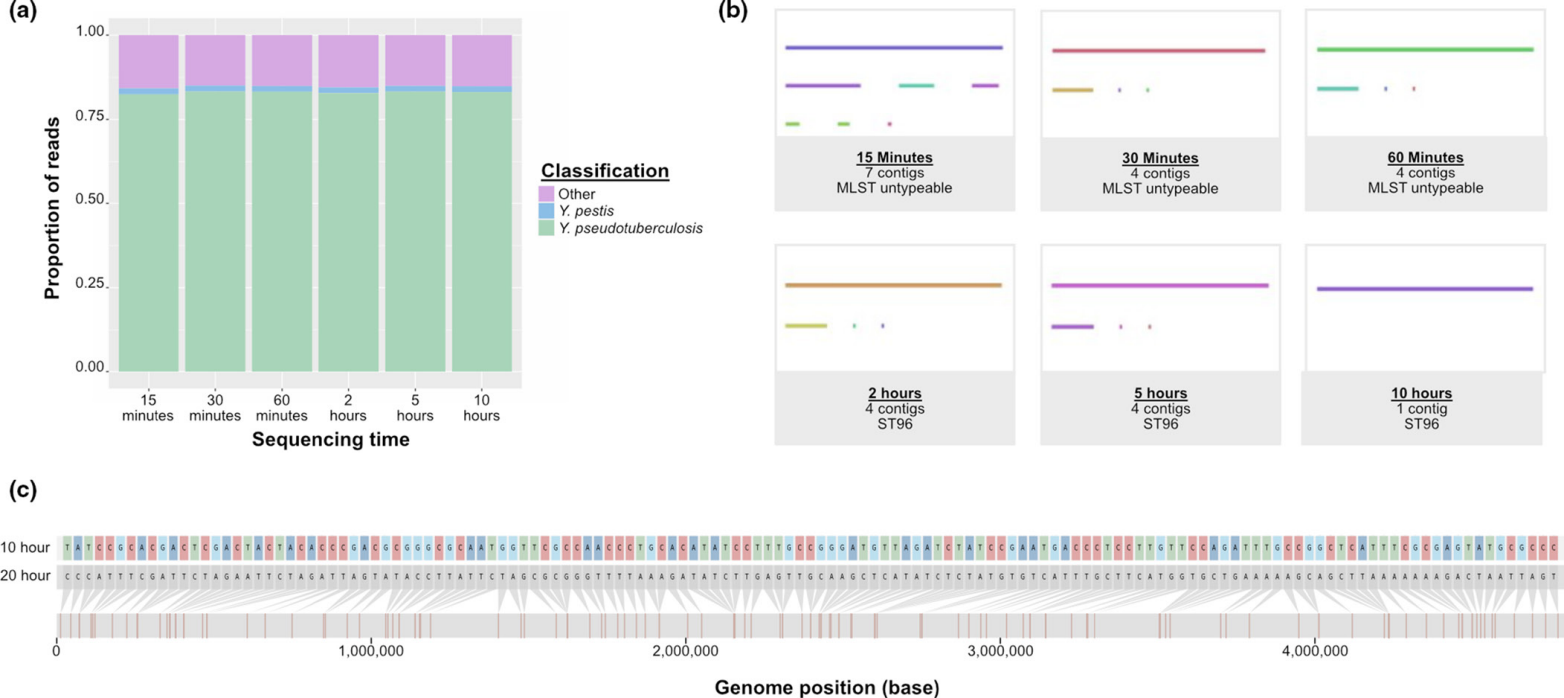
Retrospective analysis of generated FAST5 files. (**a**) Stacked histogram showing proportion of reads in each time point classified as either *Y. pseudotuberculosis, Y. pestis* or other. Total number of reads for each time point is listed on top of each histogram. Image generated using ggplot2 [19]. (**b**) Graphical representation of polished long-read assembly status and MLST result of the six time points used in the retrospective analysis. Image generated using Bandage version 0.81 [20]. (**c**) Instances of the 144 identified ‘SNPs’ between the assembly generated from 10 h of sequencing (top) and the initial assembly (middle) with positions scattered across the entire assembly (bottom). Image generated using snipit (https://github.com/aineniamh/snipit).

Nanopore sequencing on a MinION (Oxford Nanopore Technologies) was performed for 20 h with a ‘two-pronged’ approach to gather multiple lines of evidence at the reads and assembly level (Fig. 1a; Supplementary File 1, available in the online version of this article). Of the 336 622 trimmed reads, 81.7 % were classified as *Y. pseudotuberculosis* as opposed to 1.74 % as *Y. pestis* (Fig. 1b). Assembly resulted in in a single, circular 4.79 Mb contig. *In silico* Multi Locus Sequence Typing (MLST) against the McNally MLST scheme returned a *Y. pseudotuberculosis* associated sequence type (ST) of ST96 [1]. In addition, core genome MLST (cgMLST) showed that this isolate clustered together with other *Y. pseudotuberculosis* genomes (Fig. 1c, Supplementary File 2). The entire process, which took approximately 29 h (20 h of sequencing and 9 h of downstream offline base-calling and analysis), indicated that the isolate was *Y. pseudotuberculosis *. Retrospective analysis showed that data generated within 2 h would return the same results on both the read and assembly level. (Fig. 2). A contiguous assembly, albeit 59 bases shorter, could be generated from reads 10 h into sequencing with genomic difference localised to 141 ‘SNPs’ (Fig. 2), 446 insertions and 456 deletions. Confirmatory reference biochemical tests were subsequently performed which yielded results expected for *Y. pseudotuberculosis* (Table 1). Concordant genomic and phenotypic results allowed for the reclassification from *Y. pestis* to *Y. pseudotuberculosis *.

**Table 1.** Biochemical test results for the sample along with expected results for both *Y. pseudotuberculosis* and *Y. pestis
*

		Expected results^[*]^ for	
Biochemical test/substrate name	Result	* Y. pseudotuberculosis *	* Y. pestis *
Acetate	−	−	−
Adonitol	−	−	−
Arabinose	+	+	+
Arginine	−	−	−
Catalase	+	+	+
Cellobiose	−	−	−
Deaminase	−	−	−
Dulcitol	−	−	−
Esculin	+	+	+
Glucose fermentation	−	−	−
Hydrogen sulfide	−	−	−
Indole	−	−	−
Inositol	−	−	−
Lysine	−	−	−
Malonate	−	−	−
Mannitol	+	+	+
Melibiose	+	+	−
Methyl-Red	+	+	+
α-Methyl-d-glucoside	−	−	−
ONPG	+	+	+
Ornithine	−	−	−
Oxidase	−	−	−
Raffinose	−	−	−
Rhamnose	+	+	−
Salicin	−	−	+
Simmons Citrate	−	−	−
Sorbitol	−	−	−
Sucrose	−	−	−
Trechalose	+	+	+
Urease	+	+	−
Voges-Proskauer	−	−	−
Xylose	+	+	+

"*"Expected results were obtained from reference textbooks [21, 22].

## Discussion

Within the *Yersinia* genus, three species (namely *Y. pestis *, *Y. pseudotuberculosis* and *Y. enterocolitica *) are associated with human infections. While *Y. pseudotuberculosis* and *Y. enterocolitica* are both recognised enteric pathogens, *Y. pseudotuberculosis* is genomically more similar to *Y. pestis *, the causative agent of plague with the divergence from *Y. pseudotuberculosis* estimated within the last 5 700 years [2]. Indeed, a large portion of genes present in *Y. pseudotuberculosis* shared greater than 97 % nucleotide identity with their respective counterparts in *Y. pestis* [3]. This genomic similarity has implications on bacteria classification with MALDI-TOF misclassification previously documented [4, 5].

As plague is not endemic in Australia, the discordant MALDI-TOF classification triggered additional investigations for confirmation given significant implications for public health due to the incursion of a highly infectious agent into a non-endemic geolocation, and the safety of exposed healthcare staff. Human-to-human transmission has been reported from cases of pneumonic plague with household contacts and health-care workers at risk [6] while laboratory exposures can occur when *Y. pestis* is unknowingly handled outside of its recommended containment (i.e*.* PC3 in Australia). Here we employed Nanopore sequencing to provide rapid high-resolution identification.

Long read sequencing technologies have a relatively higher error rate than their short read sequencing counterparts. While we showed that taxonomical classification using long reads could identify *Y. pseudotuberculosis *, the genetic relatedness [2, 3] between *Y. pseudotuberculosis* and *Y. pestis* could have easily influenced our results if we solely relied on the sequencing reads. As such, the genome was assembled and long-read polished to minimise errors and two assembly-based *Yesinia* typing schemes [1, 7] were performed. Both schemes are capable of discriminating between *Yersinia* species and our typing results supported our initial read level taxonomical classification. Thus, the multiple lines of genomic evidence generated from sequencing read classification, MLST [1] and cgMLST [7], were instrumental for the discrimination between *Y. pseudotuberculosis* and *Y. pestis *. Our ‘two-pronged’ approach, which cumulatively took 29 h from library preparation to final results, was influenced by both limitations in our sequencing setup (lower end graphics processing unit and healthcare network firewall) and our conservative approach to capture enough data to assemble the entire genome. While the turnaround time was faster than traditional reference biochemical tests for *Yersinia* (up to 48 h), rapid identification of security-sensitive pathogens with 2 h of Nanopore sequencing have been reported [8]. Subsequent retrospective analysis showed that data generated as early as 2 h would have been enough to yield concordant species identification and typing results, further highlighting the utility of rapid identification for high-risk pathogens.

Infection with *Y. pseudotuberculosis* most commonly manifests as self-limiting acute gastroenteritis, which can lead to mesenteric lymphadenitis due to translocation to the lymphoid tissues. However, in immunocompromised patients this can also lead to invasion of deep tissue and blood, a process mediated by a suite of virulence factors [9]. A key virulence factor, the virulence plasmid pYV [10], was not detected in our analysis. This plasmid, also present in both *Y. pestis* (designated as pCD1) and *Y. enterocolitica *, harbours the genes that encode a Type 3 Secretion System (Ysc injectisome) and its associated secreted proteins (Yops) which elucidate an immunomodulatory response when secreted, allowing for survival and replication in host lymphoid tissues [10, 11]. While traditionally used to distinguish between pathogenic and non-pathogenic *Yersinia *, pYV-negative *Y. pseudotuberculosis* have also been isolated from clinical samples [12–14] and *in vitro* loss has been described [12, 14]. Nevertheless, we endeavoured to recover the plasmid-positive colonies on Tryptic-soy agar supplemented with Congo-red [15]. No pigmented colonies were obtained after multiple attempts.

While the utilisation of Nanopore sequencing can be advantageous for rapid identification of security sensitive pathogens, we acknowledge that there could be barriers for its uptake. One such barrier could be pre-existing guidelines [16], where immediate utilisation of genome sequencing is not currently recognised as part of proper procedure. Another notable limiting factor is infrastructure as sequencing devices (regardless of sequencing technology) require IT resources and firewall settings which may either not be readily accessible or compliant with cyber security policies, especially for facilities that are not currently set up for genomic analysis.

In conclusion, an in-depth investigation of a *Y. pseudotuberculosis* isolate from a sterile site that was initially misclassified as *Y. pestis* illustrated a role of rapid genome sequencing in rapid identification of security sensitive agents. While confirmatory biochemical tests are often utilised following discordant first-line classification between *Y. pseudotuberculosis* and *Y. pestis,* whole genome sequencing, especially on platforms capable of rapid turnaround time, should be considered. Indeed, rapid sequencing would allow for timely intervention for all individuals exposed and guide the appropriate infection control and public health actions if the inverse were to occur.

## Supplementary Data

Supplementary material 1
