# Supplementary material for: Case report: a genomics-guided reclassification of a blood culture isolate misassigned by MALDI-TOF as Yersinia pestis
Source: Access Microbiol. 2022 Oct 3;4(10):acmi000422. doi: 10.1099/acmi.0.000422 (PMC9675175; doi:10.1099/acmi.0.000422)
Supplement: Supplementary material 1 [file acmi-4-422-s001.pdf]

Supplementary File 1: Methods for genomic analysis

DNA extraction and sequencing

An entire plate of colonies growing on chocolate agar was sampled and DNA was extracted using the QIAamp DNA Blood Mini Kit (Qiagen) according to manufacturer's instructions under physical containment laboratory level 3 (PC3) biosafety conditions. Decontaminated DNA extraction tube was handled outside of PC3 conditions and DNA libraries were prepared using the rapid barcoding kit (Oxford Nanopore Technologies plc, SQK-RBK004) with an input DNA of 381.08 ng. Nanopore sequencing was performed on the MinION platform and sequencing was ran for 20 hours.

Base-calling and long read quality control

The sequencing run generated 510,540 reads with a mean read length and read length N50 of 5,064.5 kb and 9,542 kb respectively. Base-calling (HAC mode) and demultiplexing was performed post sequencing using Guppy[1] version 3.45+fbfbfb on a workstation equipped with an 8 GB NVIDIA graphics card. Adaptors and reads with middle adaptors were trimmed using Porechop[2] version 0.2.4. Filtlong[3] version 0.2.0 was used to exclude the worst 10% of adaptor-removed reads and reads that are shorter than 1 kb. Trimming resulted in 336,622 trimmed reads with a mean read length and read length N50 of 7.1 kb and 10.1 kb respectively.

[1] https://community.nanoporetech.com/downloads (requires Nanopore login credentials)
[2] https://github.com/rrwick/Porechop
[3] https://github.com/rrwick/Filtlong

Species classification and visualisation

Taxonomical classification was performed of the trimmed reads using Kraken2 version 2.2.1 [1] against the MiniKraken_V2 database. Kraken2 output was converted into a tabular report using "kraken-report" and visualised using Pavian version 1.0 [2].

Long read assembly, polishing, MLST and cgMLST

Trimmed reads were assembled using Flye version 2.9-b1768 [3] and assembly graphs were visualised using Bandage version 0.81 [4]. The same trimmed reads were subsequently used to polish the assembly using Minimap2 version 2.17-r941 [5] and Racon version 1.4.11 [6] for four iterations. The Racon-polished assembly was polished further with the trimmed reads using Medaka[4] version 1.2.1. *In silico* Multi Locus Sequence Typing (MLST) was performed using mlst[5] version 2.19.0 against the McNally MLST scheme [7] Core-genome MLST (cgMLST) was performed by adapting a *Yersinia spp*. scheme [8] in chewBACCA version 2.5.5 [9]. A minimum spanning tree of the cgMLST was generated Grapetree version 1.5.0 using the MSTree V2 algorithm [10]. Complete genomes of pathogenic *Yersinia* species used to populate the cgMLST analysis are listed in Supplementary File 2.

Retrospective analysis

Based on time stamps of individual FAST5 files, data generated within 15 minutes, 30 minutes, 60 minutes, 2 hours, 5 hours and 10 hours were base-called, trimmed and subjected to the same read level and assembly level analysis. When required, contigs were manually reordered according to the initial assembly using the "Contig Reordering" tool in Artemis version 18.1.0 [11]. Comparative genomic analysis to interrogate genome organisation and synteny was

[4] https://github.com/nanoporetech/medaka
[5] https://github.com/tseemann/mlst (--scheme yersinia)

performed using BLASTN and visualised using Artemis Comparison Tool version 18.1.0 [12]. Further genomic comparisons to interrogate insertions, deletions and “SNPS” between assemblies was performed using snippy[6] version 4.6.0 with the initial assembly used as reference.

[6] https://github.com/tseemann/snippy

Supplementary File 2: Genomes used for cgMLST analysis

GenBank assembly accession	Genbank Assembly name	Enterobase accession	Enterobase strain name	*Yersinia* species	Assembly status
GCF_000009345.1	ASM934v1	-	-	*Y. enterocolitica*	Complete
GCF_000192105.1	ASM19210v1	-	-	*Y. enterocolitica*	Complete
GCF_000597945.1	ASM59794v2	-	-	*Y. enterocolitica*	Complete
GCF_000834195.1	ASM83419v1	-	-	*Y. enterocolitica*	Complete
GCF_000834735.1	ASM83473v1	-	-	*Y. enterocolitica*	Complete
GCF_000968115.1	ASM96811v1	-	-	*Y. enterocolitica*	Complete
GCF_001304755.1	ASM130475v1	-	-	*Y. enterocolitica*	Complete
GCF_001305635.1	ASM130563v1	-	-	*Y. enterocolitica*	Complete
GCF_001708575.1	ASM170857v1	-	-	*Y. enterocolitica*	Complete
GCF_001708595.1	ASM170859v1	-	-	*Y. enterocolitica*	Complete
GCF_001708615.1	ASM170861v1	-	-	*Y. enterocolitica*	Complete
GCF_001708635.1	ASM170863v1	-	-	*Y. enterocolitica*	Complete
GCF_016727765.1	ASM1672776v1	-	-	*Y. enterocolitica*	Complete
GCF_016727905.1	ASM1672790v1	-	-	*Y. enterocolitica*	Complete
GCF_900637005.1	46582_C01	-	-	*Y. enterocolitica*	Complete
GCF_901472495.1	32868_C01	-	-	*Y. enterocolitica*	Complete
GCF_000006645.1	ASM664v1	-	-	*Y. pestis*	Complete
GCF_000007885.1	ASM788v1	-	-	*Y. pestis*	Complete
GCF_000009065.1	ASM906v1	-	-	*Y. pestis*	Complete
GCF_000013805.1	ASM1380v1	-	-	*Y. pestis*	Complete
GCF_000013825.1	ASM1382v1	-	-	*Y. pestis*	Complete
GCF_000016445.1	ASM1644v1	-	-	*Y. pestis*	Complete
GCF_000018805.1	ASM1880v1	-	-	*Y. pestis*	Complete
GCF_000022825.1	ASM2282v1	-	-	*Y. pestis*	Complete
GCF_000186725.1	ASM18672v1	-	-	*Y. pestis*	Complete

GenBank assembly accession	Genbank Assembly name	Enterobase accession	Enterobase strain name	*Yersinia* species	Assembly status
GCF_000834235.1	ASM83423v1	-	-	*Y. pestis*	Complete
GCF_000834275.1	ASM83427v1	-	-	*Y. pestis*	Complete
GCF_000834315.1	ASM83431v1	-	-	*Y. pestis*	Complete
GCF_000834775.1	ASM83477v1	-	-	*Y. pestis*	Complete
GCF_000834885.1	ASM83488v1	-	-	*Y. pestis*	Complete
GCF_000834905.1	ASM83490v1	-	-	*Y. pestis*	Complete
GCF_000834925.1	ASM83492v1	-	-	*Y. pestis*	Complete
GCF_000835005.1	ASM83500v1	-	-	*Y. pestis*	Complete
GCF_000970105.1	ASM97010v1	-	-	*Y. pestis*	Complete
GCF_001188675.1	ASM118867v1	-	-	*Y. pestis*	Complete
GCF_001188695.1	ASM118869v1	-	-	*Y. pestis*	Complete
GCF_001188715.1	ASM118871v1	-	-	*Y. pestis*	Complete
GCF_001188735.1	ASM118873v1	-	-	*Y. pestis*	Complete
GCF_001188755.1	ASM118875v1	-	-	*Y. pestis*	Complete
GCF_001188775.1	ASM118877v1	-	-	*Y. pestis*	Complete
GCF_001188795.1	ASM118879v1	-	-	*Y. pestis*	Complete
GCF_001188815.1	ASM118881v1	-	-	*Y. pestis*	Complete
GCF_001188935.1	ASM118893v1	-	-	*Y. pestis*	Complete
GCF_001693595.1	ASM169359v1	-	-	*Y. pestis*	Complete
GCF_002005285.1	ASM200528v1	-	-	*Y. pestis*	Complete
GCF_003798205.1	ASM379820v1	-	-	*Y. pestis*	Complete
GCF_009295925.1	ASM929592v1	-	-	*Y. pestis*	Complete
GCF_009295945.1	ASM929594v1	-	-	*Y. pestis*	Complete
GCF_009296005.1	ASM929600v1	-	-	*Y. pestis*	Complete
GCF_015159615.2	ASM1515961v2	-	-	*Y. pestis*	Complete
GCF_015190655.1	ASM1519065v1	-	-	*Y. pestis*	Complete

**GenBank assembly accession**	**Genbank Assembly name**	**Enterobase accession**	**Enterobase strain name**	***Yersinia* species**	**Assembly status**
GCF_015336465.1	ASM1533646v1	-	-	*Y. pestis*	Complete
GCF_015336865.1	ASM1533686v1	-	-	*Y. pestis*	Complete
GCF_015337285.1	ASM1533728v1	-	-	*Y. pestis*	Complete
GCF_015337645.1	ASM1533764v1	-	-	*Y. pestis*	Complete
GCF_015338045.1	ASM1533804v1	-	-	*Y. pestis*	Complete
GCF_015338205.1	ASM1533820v1	-	-	*Y. pestis*	Complete
GCF_000016945.1	ASM1694v1	-	-	*Y. pseudotuberculosis*	Complete
GCF_000047365.1	ASM4736v1	-	-	*Y. pseudotuberculosis*	Complete
GCF_000750315.1	ASM75031v1	-	-	*Y. pseudotuberculosis*	Complete
GCF_000834295.1	ASM83429v1	-	-	*Y. pseudotuberculosis*	Complete
GCF_000834355.1	ASM83435v1	-	-	*Y. pseudotuberculosis*	Complete
GCF_000834375.1	ASM83437v1	-	-	*Y. pseudotuberculosis*	Complete
GCF_000834415.1	ASM83441v1	-	-	*Y. pseudotuberculosis*	Complete
GCF_000834435.1	ASM83443v1	-	-	*Y. pseudotuberculosis*	Complete
GCF_000834475.1	ASM83447v1	-	-	*Y. pseudotuberculosis*	Complete
GCF_003798285.1	ASM379828v1	-	-	*Y. pseudotuberculosis*	Complete
GCF_003798305.1	ASM379830v1	-	-	*Y. pseudotuberculosis*	Complete
GCF_003798445.1	ASM379844v1	-	-	*Y. pseudotuberculosis*	Complete
GCF_003814345.1	ASM381434v1	-	-	*Y. pseudotuberculosis*	Complete
GCF_008693365.1	ASM869336v1	-	-	*Y. pseudotuberculosis*	Complete
GCF_900092345.1	YP4713	-	-	*Y. pseudotuberculosis*	Complete
GCF_900635715.1	32473_H02	-	-	*Y. pseudotuberculosis*	Complete
GCF_900635755.1	33467_B01	-	-	*Y. pseudotuberculosis*	Complete
-	-	YER_AA2486AA	CBSLAM1703 / NC00824-06	*Y. pseudotuberculosis*[a]	Draft
-	-	YER_AA2540AA	G5137	*Y. pseudotuberculosis*[a]	Draft

GenBank assembly accession	Genbank Assembly name	Enterobase accession	Enterobase strain name	*Yersinia* species	Assembly status
-	-	YER_AA0859AA	IP33038	*Y. pseudotuberculosis*[a]	Draft

[a]: *Y. pseudotuberculosis* isolates collected in Australia

References for Supplementary Files

[1] Wood DE, Lu J, Langmead B. Improved metagenomic analysis with Kraken 2. *Genome Biol* 2019;20(1):257. doi: 10.1186/s13059-019-1891-0.

[2] Breitwieser FP, Salzberg SL. Pavian: interactive analysis of metagenomics data for microbiome studies and pathogen identification. *Bioinformatics* 2020;36(4):1303-4. doi: 10.1093/bioinformatics/btz715.

[3] Kolmogorov M, Yuan J, Lin Y, Pevzner PA. Assembly of long, error-prone reads using repeat graphs. *Nat Biotechnol* 2019;37(5):540-6. doi: 10.1038/s41587-019-0072-8.

[4] Wick RR, Schultz MB, Zobel J, Holt KE. Bandage: interactive visualization of de novo genome assemblies. *Bioinformatics* 2015;31(20):3350-2. doi: 10.1093/bioinformatics/btv383.

[5] Li H. Minimap2: pairwise alignment for nucleotide sequences. *Bioinformatics* 2018;34(18):3094-100. doi: 10.1093/bioinformatics/bty191.

[6] Vaser R, Sović I, Nagarajan N, Šikić M. Fast and accurate de novo genome assembly from long uncorrected reads. *Genome Res* 2017;27(5):737-46. doi: 10.1101/gr.214270.116.

[7] Hall M, Chattaway MA, Reuter S, Savin C, Strauch E, Carniel E, *et al.* Use of whole-genus genome sequence data to develop a multilocus sequence typing tool that accurately identifies *Yersinia* isolates to the species and subspecies levels. *J Clin Microbiol* 2015;53(1):35-42. doi: 10.1128/JCM.02395-14.

[8] Savin C, Criscuolo A, Guglielmini J, Le Guern AS, Carniel E, Pizarro-Cerdá J, *et al.* Genus-wide *Yersinia* core-genome multilocus sequence typing for species identification and strain characterization. *Microb Genom* 2019;5(10). doi: 10.1099/mgen.0.000301.

[9] Silva M, Machado MP, Silva DN, Rossi M, Moran-Gilad J, Santos S, *et al.* chewBBACA: A complete suite for gene-by-gene schema creation and strain identification. *Microb Genom* 2018;4(3). doi: 10.1099/mgen.0.000166.

[10] Zhou Z, Alikhan NF, Sergeant MJ, Luhmann N, Vaz C, Francisco AP, *et al.* GrapeTree: visualization of core genomic relationships among 100,000 bacterial pathogens. *Genome Res* 2018;28(9):1395-404. doi: 10.1101/gr.232397.117.

[11] Carver T, Harris SR, Berriman M, Parkhill J, McQuillan JA. Artemis: an integrated platform for visualization and analysis of high-throughput sequence-based experimental data. *Bioinformatics* 2012;28(4):464-9. doi: 10.1093/bioinformatics/btr703.

[12] Carver TJ, Rutherford KM, Berriman M, Rajandream MA, Barrell BG, Parkhill J. ACT: the Artemis Comparison Tool. *Bioinformatics* 2005;21(16):3422-3. doi: 10.1093/bioinformatics/bti553.
